# Dyskeratosis congenita, stem cells and telomeres

**DOI:** 10.1016/j.bbadis.2009.01.010

**Published:** 2009-04

**Authors:** Michael Kirwan, Inderjeet Dokal

**Affiliations:** Barts and the London School of Medicine and Dentistry, Queen Mary University of London, UK; Barts and The London Children's Hospital, London, UK

**Keywords:** Dyskeratosis congenita, Stem cell, Telomerase, Telomere

## Abstract

Dyskeratosis congenita (DC) is a multi-system disorder which in its classical form is characterised by abnormalities of the skin, nails and mucous membranes. In approximately 80% of cases, it is associated with bone marrow dysfunction. A variety of other abnormalities (including bone, brain, cancer, dental, eye, gastrointestinal, immunological and lung) have also been reported. Although first described almost a century ago it is the last 10 years, following the identification of the first DC gene (*DKC1*) in 1998, in which there has been rapid progress in its understanding. Six genes have been identified, defects in which cause different genetic subtypes (X-linked recessive, autosomal dominant, autosomal recessive) of DC. The products of these genes encode components that are critical for telomere maintenance; either because they are core constituents of telomerase (dyskerin, TERC, TERT, NOP10 and NHP2) or are part of the shelterin complex that protects the telomeric end (TIN2). These advances have also highlighted the connection between the more “cryptic/atypical” forms of the disease including aplastic anaemia and idiopathic pulmonary fibrosis. Equally, studies on this disease have demonstrated the critical importance of telomeres in human cells (including stem cells) and the severe consequences of their dysfunction. In this context DC and related diseases can now be regarded as disorders of “telomere and stem cell dysfunction”.

## 1 Dyskeratosis congenita

First described as a discrete syndrome in 1910 [1], dyskeratosis congenita (DC) is a disease that can be pigeon-holed into a number of alternative classifications including “premature aging syndrome”, “bone marrow failure syndrome” and “cancer predisposition syndrome”, amongst others. In truth, DC is a highly heterogeneous disorder that is difficult to classify with precision. It was initially described as a mucocutaneous disorder and is still defined today largely by the presence of nail dystrophy, oral leukoplakia and abnormal skin pigmentation. The incidence of bone marrow failure is so high that this too is now considered a defining feature and the definition is continually expanding as links with other clinical and molecular features become more firmly established.

As well as presenting with a broad spectrum of clinical features, mutations in many genes are known to be causative (six have been published so far) with X-linked, autosomal recessive and autosomal dominant modes of inheritance recognised alongside sporadic cases [2]. Because of this clinical and genetic heterogeneity, multiple mechanisms have been postulated by which the abnormalities of DC might arise. However, the disease as it manifests in humans is widely considered to be due to defects in telomerase or in telomere maintenance. This is due at least in part to the nature of the affected genes – *DKC1*, *TERC*, *TERT*, *NOP10*, *NHP2* and *TINF2* – all of which are somehow implicated in telomere function. The severe implications of defects in the telomeric system in DC demonstrate the importance for telomere function in normal human health and this makes DC an excellent model for studying both the normal and abnormal behaviour of telomere biology in humans.

### 1.1 Clinical presentation

**Fig. 1:**
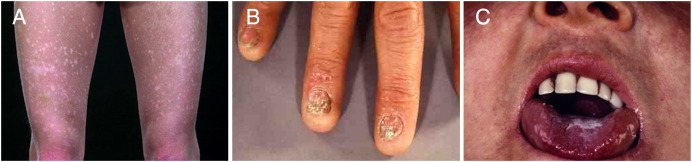
Clinical features of dyskeratosis congenita. The three mucocutaneous features that classically characterise dyskeratosis congenita are shown: (A) abnormal skin pigmentation; (B) nail dystrophy; (C) oral leukoplakia.

**Fig. 2:**
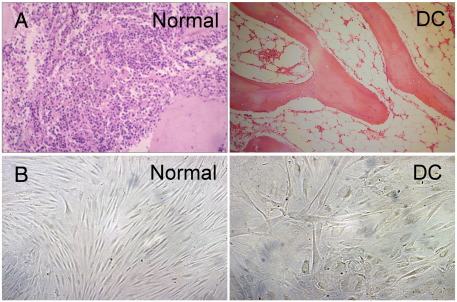
Histopathological features of dyskeratosis congenita (DC): (A) bone marrow slides show the loss of cells in an aplastic marrow, typical of dyskeratosis congenita; (B) fibroblasts grown from a patient with X-linked dyskeratosis congenita show a marked dysmorphism compared to those from a healthy individual.

As already mentioned nail dystrophy, oral leukoplakia and abnormal skin pigmentation are defining features of DC and have been referred to as the classical or common mucocutaneous triad (Fig. 1) as they are present in around 80–90% of diagnosed cases. Bone marrow failure is the other common feature that will develop in around 85% of cases and is responsible for 80% of the observed mortality [3] (Fig. 2). However, there is a plethora of other disease manifestations ranging from epiphora (excessive tears in the eyes), mental retardation, pulmonary disease (including pulmonary fibrosis and abnormal pulmonary vasculature), dental loss/caries and premature hair loss/greying to liver disease, osteoporosis and deafness [4]. Features often present in early life with skin pigmentation and nail changes usually appearing by 10 years of age followed later by mucosal leukoplakia and bone marrow failure. Epithelial tumours often begin to develop by the mid-teens and commonly arise in the gastrointestinal tract or in areas of mucosa with leukoplakia [2, 5–7]. Many of the other features such as premature loss or greying of the hair and osteoporosis are more commonly seen with aging, suggesting that premature tissue aging might be implicated as a causative factor [8]. The clinical phenotype of DC is continually expanding and so, in an effort to rationalise the diagnosis, a definition has tentatively been decided upon that is one or more of the classical mucocutaneous features combined with a hypoplastic (incompletely developed or hypocellular) bone marrow and at least two of the other somatic features [9].

One common non-clinical feature of DC is the presence of abnormally short telomeres [9,10] which is suspected as being the common, underlying cause behind most of the abnormalities. Inherited forms of the disease also demonstrate the phenomenon of anticipation [11–13] whereby successive generations of an affected family present with progressively more severe disease features and at an earlier age. This is probably due to the inheritance of short telomeres from the parent which may then also continue to shorten at an accelerated pace due to the inherited disease-causing mutation.

Several other diseases overlap with DC to the extent that they could be considered DC variants and all with the same genetic lesions. Mutations in *DKC1* and homozygous mutations in *TERT* have been shown to cause Hoyeraal–Hreidarsson syndrome (HH) [14,15], a severe multi-system disorder characterised by severe growth retardation, bone marrow failure, immunodeficiency and cerebellar hypoplasia [16–19]. Heterozygous *TERC* and *TERT* mutations have been implicated in around 5–10% of cases of aplastic anaemia (AA) [20–22], another disease of defective bone marrow defined as pancytopenia (a reduction in blood cells of all lineages) with a hypocellular marrow [23]. Heterozygous *TERC* and *TERT* mutations have also been implicated in cases of idiopathic pulmonary fibrosis (IPF) [24, 25], a chronic progressive lung disease with irreversible fibrosis leading to respiratory failure in most cases within 5 years [26].

### 1.2 Genetic basis of the disease

**Fig. 3:**
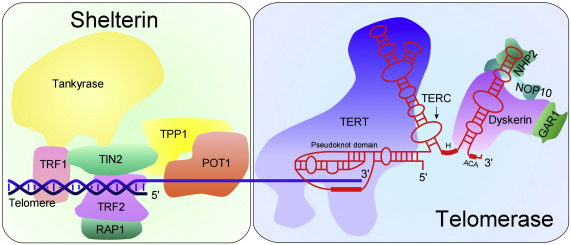
The telomerase and shelterin complexes. TERT, TERC, dyskerin, NOP10 and NHP2 in the telomerase complex and TIN2 in the shelterin complex have been shown to be mutated in cases of dyskeratosis congenita. Only around 50% of cases have been characterised at the molecular level and the other molecules shown (as well as other, unknown molecules) may yet prove to be implicated in the disease. Image derived from data in the telomerase database [93] and Cristofari et al. [94].

At the genetic level, DC is almost as heterogeneous as it is in its clinical presentation. Mutations directly implicated as causing DC have been identified in the genes *DKC1*, *TERC*, *TERT*, *NOP10*, *NHP2* and *TINF2*. The first five of these genes all encode components of the telomerase holoenzyme while *TINF2* encodes a component of the telomere shelterin complex (Fig. 3).

Mutations in *DKC1* cause the X-linked form of DC [27]. This gene encodes the nucleolar protein dyskerin which has a dual role as a pseudouridine synthase via its TruB domain, homologous to that of pseudouridine synthases in bacterial TruB proteins, yeast Pus4p and the yeast dyskerin homologue Cb5fp [28], and as an RNA-binding protein via its PUA domain which is predicted to play a role in binding H/ACA and telomerase RNAs [29,30]. In humans, functional analyses have revealed multiple roles for dyskerin, including ribosomal (r)RNA processing, ribosomal subunit assembly and centromere and microtubule binding [31–33]. Most disease-causing mutations found in human dyskerin are clustered around the PUA domain [30], suggesting that disease arises from disturbed RNA binding. Samples from X-linked DC patients also show reduced telomerase activity and reduced TERC accumulation [34,35].

TERC is the 451 nucleotide RNA component of telomerase that acts as a template for the addition of TTAGGG repeats that are added to chromosome ends to form telomeres. Mutations in *TERC* are a major cause of AD–DC [36]. Most of the *TERC* mutations identified are located in the pseudoknot domain which contains the RNA template; although large 5′ and 3′ end deletions and several other point mutations have been identified. The resulting clinical phenotype can vary from AA to DC, myelodysplasia or paroxysmal nocturnal haemoglobinuria [9,20,24,37–41].

Telomerase reverse transcriptase (TERT) is the enzymatic component of telomerase responsible for transcribing the TERC template into the TTAGGG repeat at telomere ends. *TERT* mutation has been implicated in AD-DC [12,13], AR-DC [15], HH [15], AA [21,22] and IPF [25] and while many mutations have severe effects on enzyme activity, in some cases the reduction is relatively mild, the mutation is intronic and/or the allele does not always segregate fully with the disease. For this reason, some *TERT* mutations can be considered as “risk factors” rather than directly disease-causing.

Several other proteins also associate with TERT, TERC and dyskerin in vivo, including the small nucleolar proteins NOP10, NHP2 and to a lesser extent GAR1 [42]. Biallelic mutations in both *NOP10* and *NHP2* have been implicated in AR-DC, albeit in very rare instances and, like dyskerin mutations, have been linked with reduced TERC levels in DC patients [43,44].

While all these molecules are part of the telomerase complex, dyskerin, NOP10*,* NHP2 and GAR1 are also integral components of the H/ACA class of small nucleolar ribonucleoprotein particles (snoRNPs) that bind small nucleolar RNAs (snoRNAs) [45]. These H/ACA snoRNPs guide the site-directed pseudouridylation of target RNAs and process ribosomal RNA and so are essential for ribosome biogenesis, pre-mRNA splicing and translation as well as telomere maintenance. As already mentioned, dyskerin is a pseudouridine synthase while NOP10, NHP2 and GAR1 are necessary for maintaining the stability of the H/ACA RNP complex and for pre-rRNA processing and cells lacking these proteins are defective in pre-rRNA pseudouridylation [46–48]. This, coupled with data from early animal models (see below) has led to the hypothesis that defects in dyskerin lead to DC via a pathway of aberrant ribosomal biogenesis and function, rather than via a telomerase defect [27, 49]. However, evidence from studies of DC in humans as well as in vitro studies on human cells, strongly implicates telomerase and telomeres in the fundamental processes leading to DC and there is considerable evidence showing normal functioning of pseudouridylation and rRNA precursor processing in human cells with *DKC1* mutations [35,50].

GAR1, like dyskerin, NOP10 and NHP2 is essential for pseudouridylation and ribosome biogenesis [48]. However, while GAR1 does associate with telomerase in vivo it does not appear to co-purify from human cells with the assembled telomerase complex [42], and knock-down studies of this molecule suggest that its absence has no discernable effect on TERC accumulation [44], suggesting that its association is not required for catalytic activity. It is worth noting that, at the time of writing, no DC-causing mutations have yet been identified in the *GAR1* gene despite over 100 index cases having been screened in our lab alone, further hinting that DC is primarily a disease of defective telomere biology.

*TINF2* is the only gene so far implicated in DC that does not directly involve the telomerase complex. It codes for the protein TIN2, a component (along with the proteins TRF1, TRF2, Rap1, TPP1, and POT1) of the telomere shelterin complex that protects telomere ends [51]. *TINF2* mutations cause AD-DC [52] in around 11% of all cases of DC and in some cases HH [53]. While *TINF2* is the first DC-causing gene identified outside of the telomerase complex, it is still essential for telomere maintenance. While other forms of DC are thought to cause increased telomere attrition through a reduction in TERC levels or reduced enzymatic activity, TIN2 mutations are thought to lead to more direct degradation of the telomere either by leaving the telomere end open to non-specific degradation or by preventing it from “opening-up” for telomerase to bind, although the mechanism has yet to be fully elucidated. Although there are some exceptions, patients with *TINF2* mutations tend to have very short telomeres — in fact their telomeres are generally much shorter at a much earlier age than patients with DC caused by any of the other known genes. Furthermore, almost all known cases of *TINF2*-related DC are de novo, suggesting that this form of DC is far more severe with presentation in the first few years of life in the first generation. The de novo presentation also hints that abnormally rapid telomere loss within these patients is responsible for the disease, rather than the inheritance of short telomeres from an asymptomatic parent.

## 2 Dysfunction of telomerase and telomere maintenance

### 2.1 Early animal models suggested a ribosomal defect

Data from early animal models of X-linked DC initially suggested a ribosomal defect in the disease. The first mouse model created was hypomorphic for the mouse *DKC1* homologue, *Dkc1*, resulting in a four-fold reduction of *Dkc1* mRNA expression in males and two-fold in females [54]. Despite having telomeres of a normal length, within the first generation it exhibited several of the features of DC as seen in humans including dyskeratosis of the skin, mild anaemia and a propensity to develop tumours — albeit, with a much greater incidence (50%) and different organ profile to humans (e.g. mammary glands and kidneys). Mouse *Terc* levels and telomerase activity were reduced and telomeres did shorten in successive generations, but long after the appearance of disease features. There was, however, clear evidence of reduced pseudouridine levels and slower rRNA processing. Deletion of *Dkc1* has been shown to be embryonically lethal if induced during early embryogenesis [55]. *Dkc1*^−^ hemizygous males are non-viable and though female *Dkc1^+/−^* carriers are viable they are unable to pass on the deleted gene. Mice completely lacking telomerase activity have been generated and are viable in the first few generations [56,57]; therefore a telomerase defect is unlikely to result in this loss of viability. Two *DKC1* mutations found in humans (Ala353Val and Gly402Glu), when introduced into mouse embryonic stem cells, caused a defect in overall pseudouridylation, a decrease in the rate of pre-rRNA processing and a decreased accumulation of several H/ACA snoRNAs [58]. The Ala353Val mutation however, did lead to severe destabilisation of *TERC*, a reduction in telomerase activity, and a continuous shortening of telomeres with increasing numbers of cell divisions in culture.

Models in other species also implicate telomere-independent dysfunction associated with dyskerin: partial loss-of-function mutations in the *Drosophila DKC1* homologue gene *mfl* result in reduced body size, developmental delay, reduced female fertility and morphological abnormalities in the ovaries characteristic of apoptosis [59]. In *Drosophila*, telomeres are not maintained by telomerase but by a transposon-mediated mechanism [60] and so these defects are likely to be due to some telomere-independent function of *mfl*. Loss of global pseudouridylation due to mutations in the yeast *DKC1* homologue gene *Cbf5p* results in a slow-growth phenotype and, although the cells remain viable at 25 °C, they display severe growth defects [61].

### 2.2 Recent mouse models implicate telomerase and telomeres

Despite early animal models implicating the non-telomeric role of *DKC1* mutations in disease, a more recent mouse model that directly disturbs telomere maintenance displays many of the features of DC, directly implicating telomerase and telomere maintenance in development of the phenotype. Mice null for the shelterin component POT1b were created that were smaller and had a reduced body weight, became prematurely infertile, had increased apoptosis in cells of the small intestine, hyperpigmentation of the paws, snout, ears, and tail and around 25% developed nail abnormalities. When crossed with mice deficient for the mouse *TERC* homologue *mTR*, *POT1b^−/−^ mTR^+/−^* mice were generated that showed a significant reduction of telomere length, developed a severe and progressive bone marrow failure and died at around 4–5 months of age [62].

Evidence from another mouse model shows that cells with a *Dkc1* mutation observed in human cases of DC suffer a growth disadvantage that may be due to activation of a DNA damage response that, while acting via telomerase, is independent of telomere length [63]. Female mice, like humans and other mammals, undergo a process of inactivation of half their X-chromosomes in early life which is normally random [64–67]. In this study, female mice with exon 15 deleted from *Dkc1* (*Dkc1*^Δ15^) showed a skewed X-inactivation pattern for haematopoietic cells expressing the mutant allele, something already observed in female DC patients [68]. In spleen cells of 3 week old mice, normal and mutant dyskerin proteins were present in roughly equal proportions, but by 22 weeks the mutant dyskerin represented only 16% of the total pool, suggesting a growth disadvantage for the cells expressing the mutant protein. When *Dkc1*^*Δ15/+*^ females were crossed with *Terc*^−/−^ or *Tert*^−/−^ mice, this skewing was not apparent in the telomerase negative progeny, suggesting that the mechanism is telomerase-dependent. However, since this was observed in mice in the first generation before their telomeres became unduly shortened, it appears to be independent of telomere length. Treatment with etoposide, a topoisomerase inhibitor that induces double-stranded breaks in DNA, revealed a markedly increased response to DNA damage in mutant cells with an increased localisation of DNA damage foci at the telomeres. The increased response to DNA damage would presumably lead to an increase in cell-cycle arrest and apoptosis. This telomere length-independent mechanism could thus explain the presence of DC-like features in the early mouse model within the context of normal length telomeres.

Ultimately, regardless of the data in animal models, there is a wealth of data from human study implicating telomerase and telomeres in DC and all the genes seen to be mutated in cases of DC are, without exception, directly implicated in telomere biology.

### 2.3 Telomerase mutations and DC

The molecular mechanism of *TERC* mutations appears to be either to reduce RNA accumulation/stability or cause a telomerase catalytic defect. *TERC* and *TERT* mutations have both been shown to adversely impact telomerase enzymatic function through in vitro assays such as the telomerase repeat amplification protocol [69] and in vitro studies reconstituting telomerase activity with a mix of wild type and mutant *TERC* suggest that heterozygous mutations can cause disease though haplo-insufficiency [70]. As yet, the mechanism by which mutations in *DKC1*, *NOP10* and *NHP2* affect telomerase has yet to be directly elucidated — hence the continuing debate over the role of ribosome biogenesis in disease. However, some evidence does indirectly implicate telomerase. For example, in cases of *DKC1*, *NOP10* and *NHP2* mutation, there is a marked decrease in the steady-state level of TERC within the cell [34,43,44,71], suggesting destabilisation of the telomerase complex. Short telomeres are also a universal feature of DC patients including those not associated with *TERC* or *TERT* mutations [9,10,34,72]. In fact, critically short telomere length in lymphocytes has been shown to be diagnostically discriminative for DC in around 90% of cases in one study [10].

That *DKC1* mutant cells might be subject to early cell cycle arrest is supported by evidence of an increase in apoptosis in such cells, rather than a proliferative defect. This is apparent in B lymphocyte cell lines which can be cultured over many population doublings. Compared to lines from unaffected individuals, there is a sharp increase in the apoptotic fraction of cells carrying a dyskerin Tyr66Ala mutation as the number of population doublings increases [50].

### 2.4 Telomere shelterin complex mutations

The finding of *TINF2* mutations in DC is perhaps one of the most critical in confirming the role of telomeres in the disease. In telomerase positive cells such as the HeLa tumour cell line, siRNA-mediated knock-down of TIN2 or deletion of significant portions of the N-terminus of the protein leads to an *increase* in telomere length [73,74]. This is because TIN2 appears to up-regulate the effect of the poly (ADP-ribose) polymerase (PARP) tankyrase 1 (also known as TIN1) by stabilising the formation of a TIN2–tankyrase 1–TRF1 complex. This in turn prevents binding of TRF1 to the telomere end. Normally, when TRF1 binds to the telomere it recruits another protein, POT1, and this complex blocks access to telomerase, so by preventing TRF1 binding the telomere is left open for enzymatic extension. In the case of amino acid mutations in TIN2 they might act by the same mechanism as a knock-down and leave telomere ends permanently unprotected in telomerase negative somatic tissues and thereby subject them to non-specific degradation. Alternatively, they might prevent the TIN2–tankyrase–TRF1 interaction, leaving TRF1 to fully bind to the telomere and prevent any telomere-lengthening activity during the period of rapid cell growth in embryogenesis and early life. Obviously, the close interaction of the shelterin components necessary for correct control of telomere maintenance suggests that shelterin molecules other than TIN2 may well be implicated in some of the approximately 50% of uncharacterised cases of DC.

## 3 The stem cell defect

Having established that the likely primary cause of DC is a defect in telomerase or telomere maintenance, how does this translate into disease? In embryonic, foetal and early childhood development, all tissues experience a high proliferative demand. In the context of impaired telomere maintenance, this could lead to exaggerated telomere attrition, in excess of that to be expected in healthy individuals. Telomeres would be expected to shorten in telomerase negative tissues throughout subsequent life at the normal rate but, having been severely shortened earlier on, would reach critically short lengths much earlier than normal. This would explain the features of premature aging as tissues senesce or apoptose prematurely. In highly proliferative tissues, this effect would be greatly exaggerated and these tissues would be expected to show the most severe disorders, hence the prevalence of pathologies in the bone marrow, gut and skin. Telomerase is not active in most somatic tissues and therefore a disease of defective telomerase would be expected to have its “ground zero” in a cell that would normally be telomerase positive. This leaves only a few cell types including male germ line cells, activated lymphocytes and stem cells [75]. Of these, the cell type that is likely to give rise to defects in multiple organs is the stem cell and since bone marrow failure is the cause of most DC-related mortality it is the haematopoietic stem cell (HSC) that has received the most research attention with defects having been identified in several studies. From the point of view of treatment, an important question is whether the defect is quantitative or qualitative, or a mixture of both.

### 3.1 Defective haematopoiesis

**Fig. 4:**
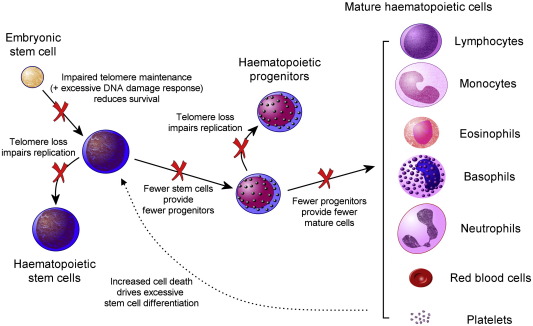
The defects in cellular proliferation and differentiation that give rise to dyskeratosis congenita. Stem cell replication is impaired and cell survival is compromised starting with the embryonic stem cell and continuing through to adult cells. This in turn drives excessive tissue-specific stem cell division, ultimately exhausting the stem cell reserve. This figure takes the haematopoietic system as an example but the principles apply equally to other tissues.

Even in healthy individuals, HSC telomeres progressively shorten with age [76] so increasing this rate of attrition is likely to severely impair the HSC pool, eventually resulting in bone marrow failure (Fig. 4). Studies of haematopoietic progenitors in DC show a clear reduction in numbers in both the bone marrow and peripheral blood [77–79] and in vitro studies of long term bone marrow culture demonstrate a quantitative deficit in global haematopoiesis [79]. Serial re-plating of DC progenitor colony forming units showed a replicative defect with the number of new colonies rapidly declining with serial passage [80]. Thus, early conclusions were of both qualitative and quantitative defects in haematopoiesis. The bone marrow CD34^+^ population is severely reduced in cases of aplastic anaemia [81] and in at least one family with a *TERC*-derived AD–DC [82] and these cells have abnormally short telomeres. However, although the number of corresponding early haematopoietic progenitors was reduced in AD-DC, their frequency within the CD34^+^ population was similar to wild type, the implication being that DC stem cells are able to develop and differentiate normally but that their replicative potential is reduced. Telomerase clearly plays a critical role in extending the replicative potential of HSCs. Those derived from *mTR*^−/−^ mice when serially transplanted showed twice the rate of telomere attrition and could be transplanted for only half as many rounds as those from *mTR*^+/+^ mice [83]. It was therefore hypothesised that replicative exhaustion of the HSC population is at the heart of the haematopoietic defect in DC. It should be noted that these studies have not yet been repeated in DC arising from the other genetic backgrounds and therefore may not apply to all genetic subtypes, where qualitative defects in haematopoiesis may exist.

If the previously mentioned study on *Dkc1*^Δ15^ mice is anything to go by, it might be the case that *DKC1* mutations cause impairment of the HSC compartment even before telomeres become critically short. If *DKC1* mutation leads to an exaggerated response to DNA damage and premature cell death then the HSC of a DC patient with a *DKC1* mutation would suffer a growth and survival disadvantage that would likely result in qualitative impairment of differentiation and development, as well as a quantitative reduction of the HSC pool.

As well as direct effects of telomerase defects within haematopoietic stem cells, telomerase dysfunction in BM stroma has an extrinsic effect on HSCs, inhibiting HSC and progenitor function and replication [84]. Telomerase deficient *mTR*^−/−^ mice showed impaired B-lymphocyte development with reduction of cell proliferation and increased apoptosis while myelopoiesis (development of new blood cells in the marrow) was accelerated. This occurred with both endogenous *mTR*^−/−^ cells and cells transplanted from a wild type *mTR*^+/+^ mouse, suggesting that the defective maturation was due to the stromal environment rather than or in addition to the intrinsic telomerase defect in the HSC. Engraftment of wild type BM cells into an *mTR*^−/−^ stromal background was also impaired in aged mice. All of this raises the possibility that transplantation and genetic therapies to correct DC defects in the HSC pool might have to overcome the additional hurdle of a detrimental environment in the marrow. However, this does not appear to be a universal feature of DC, as a previous in vitro study using stroma derived from patients with AR-DC and X-linked DC to culture wild type CD34^+^ cells showed normal levels of haematopoiesis [79]. Once again, these differences may be due to the differing genetic backgrounds that can give rise to DC and highlight the potential difficulty in developing a single treatment for such a genetically heterogeneous disorder. The possibility that a DC patient's stroma may be impaired in its ability to support HSC development also implies difficulties in using HSC transplantation as a treatment for the disease. Indeed, while allogeneic bone marrow transplantation can be successful in treating the pancytopenia in DC, fatal complications after transplantation are particularly common [85,86]. However, whether this is due to HSC engraftment or the systemic defects present in DC patients has not been conclusively demonstrated.

### 3.2 Non-haematological abnormalities

While the stem cell is at the heart of the disease, and certainly responsible for the bone marrow failure seen in DC, some of the non-haematological features and those of premature aging might also be explained by other means. Evidence from the *POT1b*^−/−^ model of DC shows similar patterns of melanin distribution to those caused by ultraviolet light exposure in human skin, which is itself a DNA damage response. This might suggest that the abnormal skin pigmentation seen in DC is a response to DNA damage [62]. The parallels between the abnormal skin pigmentation in DC and increased susceptibility to DNA damage in *Dkc1* mutant mouse epithelial cells with the type of skin pigmentation seen as a response to UV damage are intriguing and suggest a mechanism for some of the non-haematological features that does not simply rely on premature cell death.

However, exhaustion of the local stem cell pool in any affected organ is usually considered to be the most likely cause of disease features in DC. Unusually rapid turnover of cells in the lungs and gastrointestinal lining could be the driving force behind the propensity for fibrosis and tumours seen in these tissues. This would be expected to set up a positive feedback loop, with premature and thus excessive loss of adult cells driving excessive replication and differentiation in the stem cell compartment. Mutations in *TERT* and *TERC* have been directly associated with IPF [12,25,87,88] where the telomeres are short and tissue damage is focal and scattered across the parenchyma. It is therefore possible that a telomerase defect might be resulting in a failure to respond to tissue damage due to the loss of lung stem cells. IPF patients have shown some response to antioxidant treatment including increased epithelial lung fluid glutathione levels and mildly improved lung function tests [89–92], suggesting that prevention of cell damage or loss due to oxidative stress can improve the prognosis. While the same effects have not yet been examined specifically in DC-related pulmonary disease, it is reasonable to suppose that there are parallels in the pathogenesis in terms of excessive cell loss.

As yet, studies of the molecular basis of non-haematological aspects of DC are somewhat lacking by comparison to haematological studies. Further research into the areas of, for example, organ-specific dysfunction and solid tumour development are required in order to better understand the down-stream effects of telomere dysfunction on other systems and ultimately to develop a treatment to cure or ameliorate the symptoms.

## 4 Summary and future directions

**Fig. 5:**
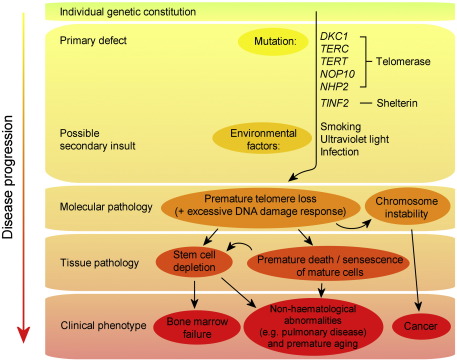
A model of dyskeratosis congenita pathology. Mutations in telomerase and shelterin components cause excessive telomere attrition and may increase the response to normal DNA damage, a process which is exacerbated by environmental stresses. This leads to premature cell death and chromosome instability which eventually either exhausts the stem cell reserve or results in haematological or non-haematological cancers.

From a basic scientific standpoint, dyskeratosis congenita can be considered a direct demonstration of the effect of dysfunctional telomere biology in humans — something that has been difficult to reproduce even in common animal models such as the mouse. In this sense it provides an excellent system for studying the complexities and functional consequences of telomere biology. Although a general model can be elucidated (Fig. 5) the broad phenotypic range, involvement of multiple genetic components and the interplay between molecular and environmental effects show that DC is a complex disorder that has yet to be fully understood and will no doubt provide further insight into both telomere biology and the complexities of human disease.

Even after many years of study and having uncovered the causative genes involved, there is still no satisfactory treatment for the disease. Further molecular studies are necessary to identify which genes are implicated in the remaining uncharacterised cases and more research is required to pin down the precise mechanisms involved in the pathogenesis of DC, especially the differences between genetic subtypes, in order for a more rational and efficacious approach to treatment to be developed. To date, the advances in the genetics of DC have led to early diagnosis (including antenatal diagnosis) and more rational use of drugs for the bone marrow failure; this includes the use of upfront oxymetholone rather than immunosuppressive therapy in patients presenting with “cryptic” DC. They have also reinforced the idea that in patients who are to undergo haematopoietic transplantation, drugs such as busulphan and radiotherapy should be avoided because of the abnormal tissue repair as a consequence of the telomere defect. Equally they have also led to a better understanding of some related diseases such as idiopathic pulmonary fibrosis and idiopathic aplastic anaemia.

Being such a rare disease it is unlikely that pharmaceutical companies would be willing to invest the vast sums necessary to develop a new drug purely for this one application. Since DC is a genetic disorder (and in the characterised cases, monogenic) it might therefore lend itself to genetic therapy. While this has been touted for some years, surprisingly little progress has been made in this area and the absence of a satisfactory animal model is a significant obstacle to the development of a therapeutic vector.

However, because of the greater understanding now achieved with regards to the molecular basis of DC, the ever-widening phenotype associated with telomerase dysfunction and the involvement of telomeres in several other disorders such as cancer and aging, DC is being studied ever more broadly and intensively and this can only be beneficial to the wider scientific community and those unfortunate enough to suffer from this rare disorder.
